# Transcriptome Analysis Revealed the Advantages of Room Temperature Preservation of Concentrated *Oocystis borgei* Cultures for Use in Aquaculture

**DOI:** 10.3390/ijms242216225

**Published:** 2023-11-12

**Authors:** Ning Zhang, Chengcheng Deng, Ting Hong, Jiajia Ren, Yulei Zhang, Feng Li, Zhongdian Dong, Zhangxi Hu, Xianghu Huang, Changling Li

**Affiliations:** 1 Lab of Algae Resource Development and Aquaculture Environment Ecological Restoration, Fisheries College, Guangdong Ocean University, Zhanjiang 524088, China; zhangn@gdou.edu.cn (N.Z.); 2112101072@stu.gdou.edu.cn (C.D.); captaintein@126.com (T.H.); rjj12138@126.com (J.R.); zhangyl@gdou.edu.cn (Y.Z.); lifeng2318@gdou.edu.cn (F.L.); zddong@gdou.edu.cn (Z.D.); huzx@gdou.edu.cn (Z.H.); huangxh@gdou.edu.cn (X.H.); 2 Guangdong Provincial Key Laboratory of Aquatic Animal Disease Control and Healthy Culture, Fisheries College, Guangdong Ocean University, Zhanjiang 524088, China

**Keywords:** *Oocystis borgei*, room temperature preservation, RNA-Seq, photosynthesis, polysaccharides

## Abstract

*Oocystis borgei*, a microalgae species employed for regulating the quality of aquaculture water, demonstrates the capacity to adsorb noxious substances, curtail the growth of detrimental bacteria, and outcompete blooming cyanobacteria. It can be concentrated by natural sedimentation and stored at room temperature, making it costless and simple to transport and use. To study the mechanism of adaptation to room temperature preservation, *O. borgei* was concentrated (1.19 × 10^7^−1.21 × 10^7^ cell/mL) and stored for 50 days at low (5 °C, LT), normal (25 °C, NT), and high (35 °C, HT) temperatures, respectively. Polysaccharide content, lipid content, cell survival, and resuscitation were evaluated. RNA-Seq was also used to examine how concentrated *O. borgei* responded to temperature. During storage, there was an increase in polysaccharide content and a decrease in lipid content, with both being significantly upregulated in the LT and HT groups. Survival and cell density were highest in the NT group. The RNA-Seq analysis revealed extensive differences in transcript levels. ATP synthesis was inhibited in the LT group due to the reduced expression of *PsaD*, *PsaE*, *PsaF*, *PsaK*, and *PsaL*. Under HT, the formation of reactive oxygen species (ROS) was facilitated by low levels of redox-related genes (*nirA*) and high levels of oxidative genes (*gdhA*, *glna*, and *glts)*. The findings suggest that storing concentrated *O. borgei* at room temperature is optimal for microalgae preservation, enhancing theoretical research in this field. Our study provides further theoretical and practical support for the development of *O. borgei* as a live ecological preparation for aquaculture microalgae ecology management.

## 1. Introduction

The recent rapid expansion of the shrimp aquaculture industry influenced the ecological balance of the aquaculture pond and resulted in a surge of diseases causing significant economic losses [1,2,3]. Microalgae plays a crucial role in the aquatic environment required for successful shrimp cultivation [4,5]. The technique of microalgae ecology control, namely adding beneficial live microalgae to establish a benevolent microalgae population and enhance the water quality in aquaculture ponds, is a successful technique for addressing the issues [6,7,8,9,10]. Live microalgae can not only be utilized as a tool to enhance the overall health and sustainability of aquaculture operations. Additionally, they constitute a crucial component of the diets of many aquaculture species [11] and various zooplankton that serve as food for the larval stages of marine fish and crustaceans [12]. Moreover, some species of live microalgae have higher nutritional value and digestibility than other alternative feeds, which is better for the development, growth, survival, and digestion of larvae of aquatic animals such as bivalve molluscs, shrimp, and sandfish [13,14,15,16]. However, due to the sensitivity of microalgae to environmental conditions, microbial contamination, and operational variables, large-scale continuous cultivation of live microalgae is time- and financially consuming (about 30–50% of total aquaculture costs) [17,18]. The culture crashes of live microalgae used to raise larval and juvenile aquaculture species at critical points in a hatchery’s operation can even be devastating to aquaculture [19]. This is the reason why concentrated microalgae were extensively studied [20,21].

Microalgae concentrates, which can be transported from central production facilities to aquaculture operations, are increasingly sought after within this market niche [19,22,23,24]. To be suitable for commercial use, microalgae concentrate should have a shelf life of at least two months and maintain a stable biochemical profile during storage [21]. Cryopreservation is currently the most widely used method [25]; however, the intricate procedure as well as the high costs make it not suitable for saving large quantities of biomass. Meanwhile, it is entirely unnecessary for long-term (year-level) preservation since concentrated microalgae are routinely recultivated in a matter of months. Normal temperature storage is more appropriate for preserving, transporting, and quickly utilizing concentrated microalgae used in aquaculture while also being more cost-effective. Currently, there are limited studies on the preservation of microalgae at room temperature because concentrated microalgae preserved at room temperature are still engaged in a low-level metabolism, such as respiration and photosynthesis, which may lead to the starvation of microalgae or the breeding of harmful bacteria [21,24,26,27,28].

*Oocystis borgei*, a kind of green algae isolated from the high-level pond of shrimp in South China [29], possesses the ability to prevent shrimp disease [30]. Directional cultivation of *O. borgei* in shrimp ponds can effectively absorb harmful inorganic nutrients such as ammonia and nitrite [21,31,32]], compete with bloom cyanobacteria [33,34], and inhibit the growth of harmful bacteria (*Vibrios*) in the cultured water and the intestine of shrimp [30,31,35]. Furthermore, *O. borgei* can be effectively collected via natural gravity sedimentation that is costless and has good maintenance of cell viability [36], and the concentrated live cultures can be further sealed in a plastic bottle and stored for at least six months at room temperature (based on long-term application practice). Any aquaculturist could easily store and transport the concentrated cultures to rapidly produce voluminous, high-quality fresh microalgae for the purpose of microalgae ecology control technology.

As indicated by the information above, preserving concentrated *O. borgei* at room temperature holds a promising future in aquaculture. However, the mechanism of long-term preservation of *O. borgei* in a concentrated state at room temperature is not clear, which hinders the further development of *O. borgei* as an ecological preparation. In this study, changes in polysaccharide and lipid contents of concentrated *O. borgei* were monitored at 5 °C (low temperature, LT), 25 °C (room temperature or normal temperature, NT), and 35 °C (high temperature, HT). After 50 days of preservation, the effect of temperature on concentrated microalgae was evaluated based on the survival rate and recovery efficacy. Furthermore, transcriptional profiles of concentrated *O. borgei* in response to various preservation temperatures were evaluated to reveal the molecular mechanisms underlying adaptation to room-temperature storage. The research findings will enhance the theoretical research on room-temperature preservation of microalgae and will promote the simple preservation of *O. borgei* for direct use in shrimp culture, thereby facilitating microalgae ecology control in the aquaculture industry.

## 2. Results

### 2.1. Effect of Storage Temperature on Polysaccharide and Lipid Contents in O. borgei

**Figure 1:**
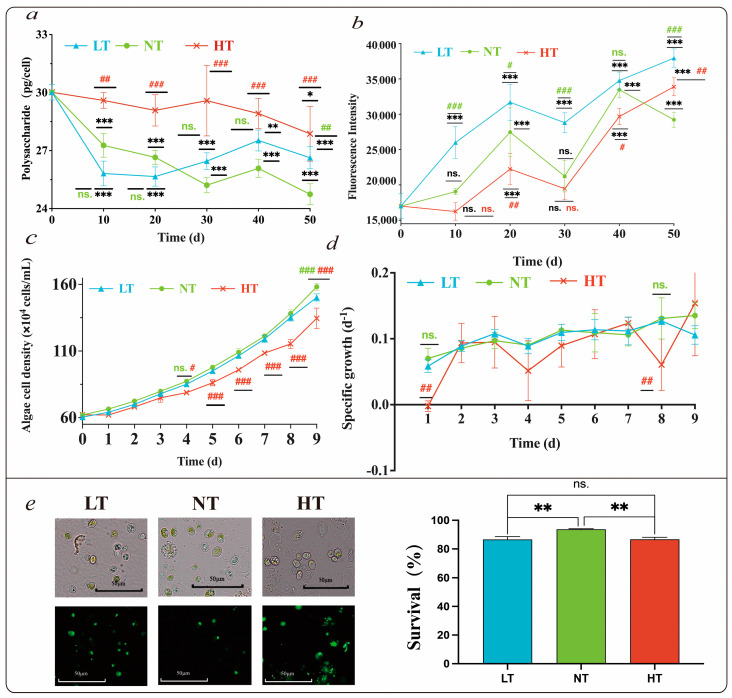
Changes in polysaccharide content (**a**), lipid content (**b**), cell density (**c**), specific growth rate (**d**), and the survival rate (**e**) of *O. borgei.* The data show that the mean value ± standard deviation (*n* = 3). *: intragroup significant difference between values and initial values at any time point (*: *p* < 0.05, **: 0.001 ≤ *p* < 0.01, ***: *p* < 0.001), and #: inter group differences, red indicates a significant difference between NT and HT, and green indicates a significant difference between LT and NT’ (#: *p* < 0.05, ##: 0.001 ≤ *p* < 0.01, and ###: *p* < 0.001), ns. indicates that the difference between the comparison groups was not significant.

The equation for the glucose standard curve was OD = 0.0054 [glucose] + 0.2603, of which R^2^ was 0.9987 (using least-square fit). The polysaccharide contents of *O. borgei* in the LT and NT groups decreased significantly during the initial 20 days (*p* < 0.001); however, remained stable from day 20 to 50. After 50 days of storage, the polysaccharide content decreased significantly by 11.24% (*p* < 0.001) and 18.54% (*p* < 0.001) in the LT and NT groups, respectively, compared to values before storage. In the HT group, the polysaccharide content of *O. borgei* was consistently higher, reaching 27.88 pg/cell on day 50, which was 18.54% lower than the initial value of 30.02 pg/cell (*p* = 0.013). The HT group showed significantly higher polysaccharide content than that of the NT group on day 10 of preservation (*p* = 0.01) and an extremely significantly higher polysaccharide content than the NT group on days 20 to 40 (Figure 1a). On day 50, the polysaccharide contents in both the HT (*p* < 0.001) and LT (*p* = 0.01) groups were significantly higher than that in the NT group.

The total lipid content showed an overall upward trend and was significantly or extremely significantly higher in the LT group than in the NT group, except on day 40. After 50 days of storage, the lipid contents in LT, NT, and HT groups increased significantly by 113.84%, 71.94%, and 99.39% compared to the initial value, respectively, and the content in the NT group was significantly higher than those in the LT (*p* < 0.001) and HT (*p* = 0.006) groups, respectively (Figure 1b).

### 2.2. Survival Rate and Resuscitation of O. borgei

After treatment with SYTOX Green, a survival rate of 93.67% was observed in *O. borgei*, which was extremely significantly higher than those in the LT (83.63%) (*p* = 0.003) and HT groups (86.68%) (*p* = 0.003) (Figure 1e). After recovery, the cell density of the NT group became significantly higher than that of the HT group on the fourth day (*p* = 0.032). From days 5 to 9, the cell density of the NT group was extremely significantly higher than that of the HT group (*p* < 0.001) and was only extremely significantly higher than that of the LT group on day 9 (*p* < 0.001). Although the cell densities of the HT group and LT group were lower than those in the NT group at the same time point during resuscitation culture, the population was restored by increasing the culture time (Figure 1c). The specific growth rate of all samples showed an upward trend 3 days before recovery and stabilized for 3–7 days (Figure 1d). The relative growth rates of the NT group on day 1 and day 8 were extremely significantly higher than those in the HT group (*p* = 0.005) and were higher than that of the LT group; however, this difference was not significant. Cell viability was lowest in the HT group, consistent with the observation that the survival rate and cell density of the HT group during storage and resuscitation were lower than those of the NT group.

### 2.3. RNA-Seq Quality Control and Unigene Functional Annotation

cDNA libraries were constructed for *O. borgei* under LT, NT, and HT storage conditions and used for transcriptome sequencing. A total of 97.97 Gb of raw data was obtained. After cleaning and quality checks, Illumina sequencing generated 97.30 Gb of clean data, with Q_20_ values between 97% and 98% and Q_30_ values between 93% and 94% (Supplementary Table S1). After de novo transcriptome assembly using Trinity, 44,737 unigenes were obtained, with a N_50_ length of 2079 bp and GC content of 59.64% (Supplementary Tables S2 and S3). Using the Nr, SwissProt, KEGG, and KOG databases, 19,910, 11,673, 17,276, and 10,151 unigenes were successfully annotated, respectively (Supplementary Tables S4–S7). Using the Nr database, the gene sequences of *O. borgei* were homologous to genes in 361 known species (Supplementary Figure S1 and Table S8). In total, 20.6% (4098) of unigenes were homologous to genes in *Coccomyxa subelipsoidea C-169*, 18.8% (3744) of unigenes were homologous to genes in *Auxenochlorella protothecoides* (Supplementary Figure S1), and 7.9% (1580) of unigenes were homologous to genes in *Chlorella variabilis.*

### 2.4. DEG Analysis

**Figure 2:**
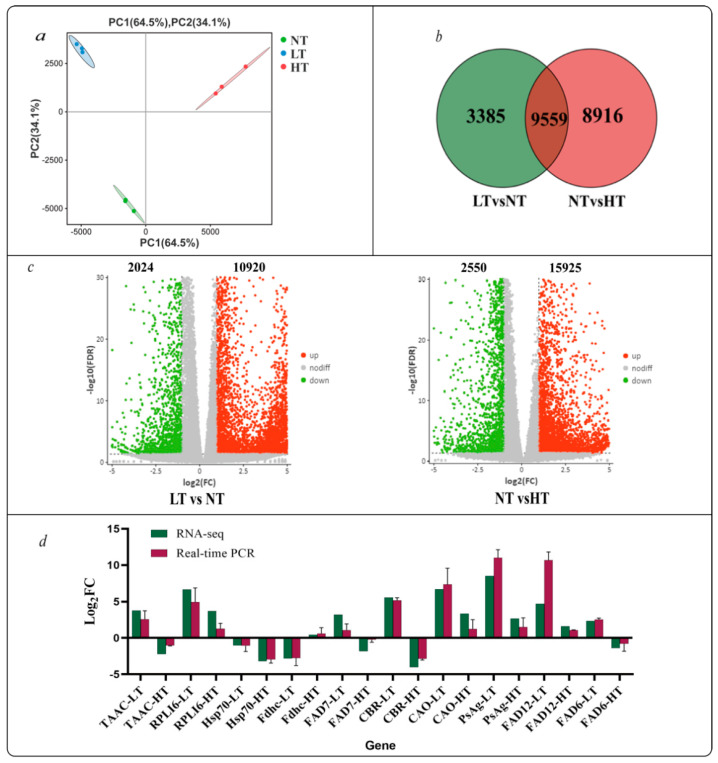
PCA (**a**) and Venn diagram (**b**) of DEGs in *O. borgei* under different preservation temperatures. (**c**) Volcano plot of LT vs. NT and NT vs. HT, “LT vs. NT” is NT normalized to LT, “NT vs. HT” is HT normalized to NT. (**d**): RT−qPCR validation of the relative expression profiles of ten genes in *O. borgei* at different storage temperatures. LT: Expression in the low temperature group relative to that in the NT group. HT: expression in the high temperature group relative to that in the NT group. TAAC: ATP carrier protein, RPLI6: 60S ribosomal protein, Hsp70: heat shock protein 70, Fdhc: nitrite transporter NAR1, FAD7: chloroplast glycerolipid omega−3−fatty acid desaturase, FAD6: omega-6−fatty acid desaturase, chloroplast isoform, CBR: carotene biosynthesis−related protein, CAO: chlorophyll an oxygenase, PsAg: photosystem I reaction center subunit V, chloroplastic, and FAD12: omega−6 fatty acid desaturase, endoplasmic reticulum isozyme 2.

A sample correlation analysis demonstrated satisfactory within-group replication and evident between-group partitioning (Figure 2a), indicating that the storage temperature has a significant impact on *O. borgei* and has potential value for further analysis. There were notably more up-regulated genes than down-regulated genes in the HT group in comparison to the LT group (Figure 2c). There were 12,944 and 18,475 differentially expressed genes (DEGs) identified in the comparison of LT versus NT (LT vs. NT) and NT vs. HT, respectively (Figure 2c; Supplementary Tables S9 and S10). A Venn diagram was employed to analyze the DEGs, revealing that 21860 DEGs were temperature-sensitive, including 9559 DEGs affected in all treatments (Figure 2b). In addition, there were 3385 and 8916 unique DEGs in LT vs. NT and NT vs. HT, respectively. Ten DEGs involved in photosynthesis and lipid biosynthesis pathways were randomly selected for RT-qPCR validation, and a melting curve analysis of each gene showed a single peak (Supplementary Figure S2). The results for all genes are consistent with the sequencing results, indicating that the sequencing data were reliable (Figure 2d).

### 2.5. GO and KEGG Enrichment Analysis

**Figure 3:**
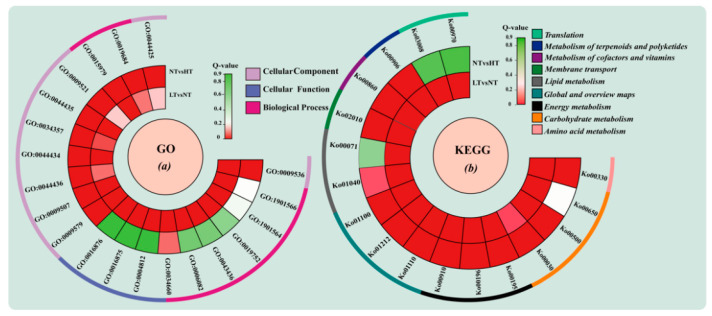
GO (**a**) and KEGG (**b**) assignment of DEGs in different temperature groups of *O. borgei.*

A GO enrichment analysis of DEGs in the LT vs. NT and NT vs. HT was performed and the top 10 significant GO terms in each comparison were used to generate maps (Figure 3a). Findings showed significant enrichment of DEGs in various GO terms, including plastid (GO: 0009536) in the cellular component category, aminoacyl−tRNA ligase activity (GO: 0004812), carbon-oxygen lyase activity (GO: 0016875), and aminoacyl-tRNA formation and compound ligase activity (GO: 0016876) in the molecular function category and ncRNA metabolic process (GO: 0034660), organic acid metabolic process (GO: 0006082), oxoacid metabolic process (GO: 0043436), and other GO terms in the biological process category in the LT vs. NT comparison. Among the top ten GO terms enriched in the NT vs. HT comparison, more GO terms were classified as cell components, including thylakoids (GO: 0009579), membrane part (GO: 0044425), and photosynthetic membranes (GO: 0034357), indicating that storage at a higher temperature causes changes in cellular structure.

To further understand the biological functions of DEGs, a KEGG enrichment analysis was performed, and the top 10 metabolic pathways significantly enriched in each comparison were selected (Figure 3b). The metabolic pathways included nine secondary pathways: amino acid metabolism, carbohydrate metabolism, energy metabolism, lipid metabolism, membrane transport, and translation. Amino acid metabolism included arginine and proline metabolism (Ko00330), and carbohydrate metabolism included three tertiary pathways: butanoate metabolism (Ko00650), starch and sucrose metabolism (Ko00500), and pentose phosphate metabolism (K000030). The global and overview maps included fatty acid metabolism (Ko01212) and the metabolic pathway (Ko01100). The four tertiary pathways of aminoacyl-tRNA biosynthesis (K00970), butanoate metabolism (Ko00650), ribosomal biogenesis in eukaryotes (Ko03008), and unsaturated fatty acid synthesis (Ko01040) were significantly enriched in the LT vs. NT group. Photosynthesis-antenna (Ko00196), photosynthesis (Ko00195), and nitrogen metabolism (Ko00910) were significantly enriched in the NT vs. HT group. The biosynthesis process of secondary metabolites (Ko01110), chlorophyll metabolism (Ko00860), and metabolic pathway (Ko01100) were significantly enriched in both comparisons, indicating differences in the physiological activities of algae cells under different storage temperatures.

## 3. Discussion

In this study, there were differences in the cell density and specific growth rate after the resuscitation of *O. borgei*, *i*ndicating a certain level of impact on cell viability during storage. Regardless of the storage temperature, *O. borgei* can recover its population density in a relatively short time after storage for 50 days. This indicates that *O. borgei i*s highly adaptable to concentrated storage in different temperature environments, providing a considerable advantage for industrial purposes. However, in terms of survival rates and recovery, room temperature was better than low and high temperatures, contradicting previous research results [21,24,37]. Our research further affirms *O. borgei’s* capacity for long-term storage at room temperature. This difference among studies may be explained by differences in the concentration of microalgae. In this study, a concentrated algae solution was used for storage, resulting in a much higher cell density than that used in normal cultivation conditions. The environmental capacity to accommodate algal cells was also higher than saturation level, thus maintaining the number of cells within the original range of values. In the absence of external factors, algal cells in a concentrated solution stored at room temperature naturally settle on the culture substrate. The microalgae at the bottom are unable to access light and nutrients and therefore enter a dormant state. However, in low or high-temperature environments, *O. borgei* needs to produce substances that confer tolerance to environmental changes, causing spores that were previously dormant to undergo physiological activities. In concentrated algae solutions, many cells are unable to access light, which hinders photosynthesis. The consumption of nutrients exceeds the supply, resulting in cell death.

Transcriptome analysis of concentrated *O. borgei* stored at different temperatures for 50 days revealed 44,737 unigenes, with a N50 of 2079 bp, which is higher than that of several previously published algal transcriptomes, such as *Chlamydomonas moewusii* (1066 bp) [38], *Chlorella* (1036 bp) [39], *Chlorella sorokiniana* (1446 bp) [40], and *Scenedesmus dimorphus* (1148 bp) [41], but lower than that of *Schizochytrium* sp. *S056* (2104 bp) [42]. This indicates that the assembly quality for *O. borgei* was relatively good. Furthermore, 20,108 unigenes were successfully annotated in the Nr, SwissProt, KEGG, and KOG databases, filling the gap in gene information for *O. borgei.* In the Nr database, 19,910 annotated unigenes were aligned to known genes from 361 species, and homology was highest with *Coccomyxa subelipsoidea C-169* [43], *Auxenochlorella protothecoides* [44], and *Chlorella variabilis* [45]. This is consistent with their close evolutionary relationships, as these species all belong to the class *Trebouxiophyceae.*

**Figure 4:**
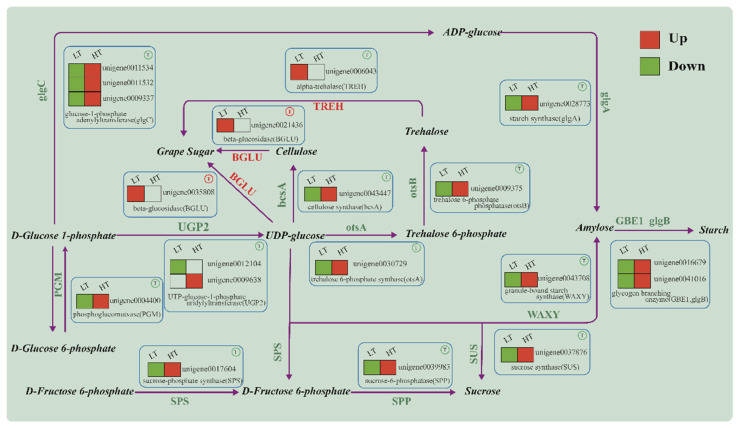
The pathway of polysaccharide synthesis of *O. borgei.*

When plants are exposed to temperatures beyond their optimal range, the structure and function of the cell membrane will undergo changes in various properties, such as fluidity, permeability, intercellular signal transduction, and antioxidant capacity [46]. Lipids and carbohydrates play important roles in plant adaptation to temperature changes, and microalgae can regulate the content and types of unsaturated fatty acids to alter the fluidity of cell membranes, while carbohydrates can enhance the tolerance of plant tissues to temperature stress [47,48]. Trehalose can protect the biofilm and protein structure of algae, ensure the activity of enzymes in adverse environments, and higher levels of trehalose can increase the temperature tolerance of algae [49,50,51]. In this study, the polysaccharide synthesis pathways were downregulated in the LT group compared to the NT group (Figure 4), suggesting that the polysaccharide synthesis pathway was suppressed. Additionally, the key cellulose hydrolase gene *BGLU* was upregulated and the trehalose hydrolysis gene *TREH* was upregulated, leading to the hydrolysis of cellulose and trehalose to fructose (Figure 4), which may explain the decrease in polysaccharides in the early stage of storage. This is contrary to the results obtained by Nagao et al. [52] and Valledor et al. [49]. Genes associated with polysaccharide synthesis were markedly upregulated in the HT group compared to the NT group. The genes included phosphoglucomutase (PGM) and UTP-glucose-1-phosphate uridylyltransferase (UPG2), which, respectively, facilitate the conversion of Glc-6-P to Glc-1-P and Glc-1-P to UDP glucose. This finding aligns with the observed rise in polysaccharide levels during HT storage, which supports Chen and colleagues’ research [48].

The response of algae to temperature stress encompasses alterations in physiological processes and biochemical components. The regulation of photosynthetic organs is crucial for microalgae to adapt to changes in environmental temperature [53,54]. Chong et al. [55] discovered that PSII-CP47 demonstrates differential expression in response to temperature stress in *Antarctic Chlorella*, with overexpression occurring at 4 °C. Additionally, during cold acclimation in the polar diatom *Fragilariopsis cylindrus*, genes encoding core proteins of PSII (*psbA* and *psbC*) are upregulated [56].

**Figure 5:**
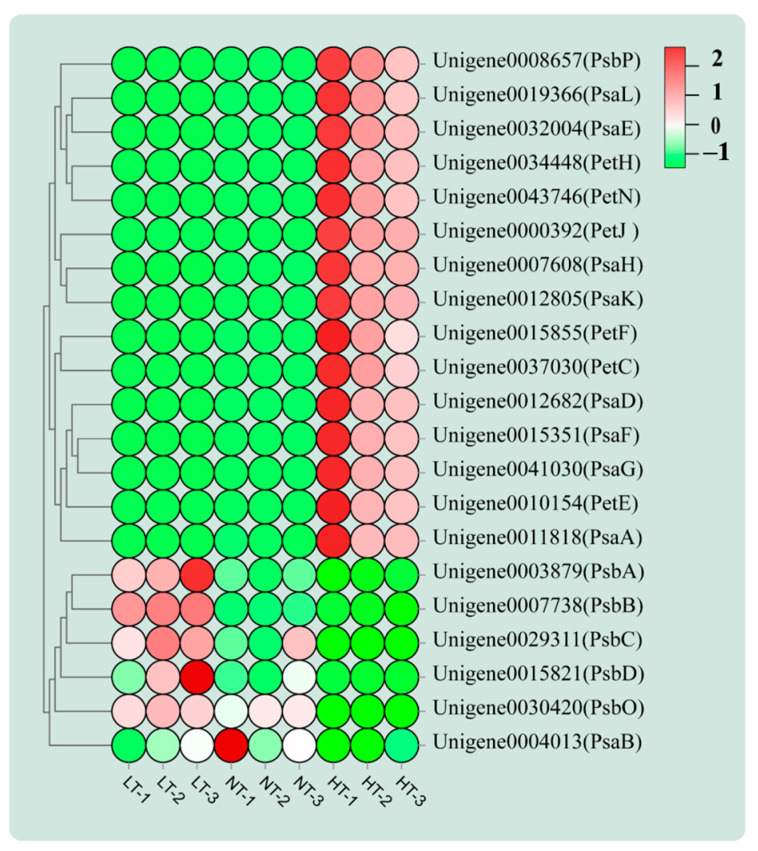
Heat map of transcript levels of photosynthesis related genes.

Photosynthesis in *O. borgei* is influenced by storage temperature. In the LT group, the expression of the genes that encode PSII D1 and D2 proteins (*PsbA* and *PsbD*) and cp47 (*PsbB*) were upregulated compared to levels in the NT group (Figure 5). This suggests that low temperatures increase the stability of the PSII reaction centre, resulting in improved internal energy transfer efficiency. An oxygen-evolving enhancergene (*PsbP*) was downregulated in the LT group (Figure 5), suggesting that oxygen release was decreased. Genes encoding phycocyanin-PC (*PetE*), ferredoxin protein (*PetF*), ferredoxin-NADP reductase protein (*PetH)*, cytochrome-b6/f complex proteins (*PetC* and *PetN)*, and cytochrome-c6 (*PetJ)*, involved in photosynthetic electron transport, were all downregulated in the LT group (Figure 5). Moreover, genes encoding P700 chlorophyll-a-apoprotein-A1 (*PsaA*) and subunit-V (*PsaG*) as well as the peripheral protein subunit genes (*PsaD* and *PsaE*) and integral membrane protein subunit genes (*PsaF*, *PsaK*, and *PsaL*) were all downregulated in PS I in the LT group (Figure 5). These findings suggest that the synthesis of structural proteins for the cytochrome-b6/f complex and the PS I reaction centre are significantly inhibited under low temperature, thereby inhibiting electron transfer. In addition, genes encoding the hydrophobic subunits (α, γ, and δ) and the subunit-a of the F-type ATPase were all downregulated in the LT group (Supplementary Figure S3a), indicating that ATP synthesis is inhibited.

In the HT group, the downregulation of *PsbD* and *PsbB* in PSII in *O. borgei* (Figure 5) indicates that the stability of the PSII reaction centre and the efficiency of the internal light energy transfer are inhibited. Additionally, the MSP gene (PsbO), which boosts oxygen evolution, was also downregulated. The P700 chlorophyll-a-apoprotein-A2 gene (*PsaB*) in PSI was also downregulated, consistent with the results of Cao et al. [57]. In contrast with the LT group, the expression levels of *PsaD*, *PsaE*, *PsaF*, *PsaK,* and *PsaL* were upregulated in HT group (Figure 5). It is important to note that the cytochrome-c6 protein (*PetJ*) in the photosynthetic electron transport chain was upregulated (Figure 5), indicating that the electron transfer process between the cytochrome b6/f complex and PSI was promoted. These findings were confirmed by RT-qPCR (Figure 2d), suggesting that they may be due to the light conditions during cultivation. Furthermore, the expression levels of subunit-a of the F-type ATPase and the hydrophobic subunits-α were upregulated, while hydrophobic subunits-δ and subunit-b of the F-type ATPase were downregulated (Supplementary Figure S3b), indicating that the transfer of H^+^ and ATP synthesis were partially inhibited. Photosynthesis was inhibited to a certain extent under both high and low-temperature stresses.

Nitrogen is an essential nutrient for microalgae growth, serving as a key component of proteins, nucleic acids, and chlorophyll. Plant nitrogen metabolism involves four reduction pathways and two oxidation pathways, with nitrate reductase and glutamine synthetase being key enzymes. Nitrate reductase activity is often used as an indicator of nitrogen metabolism, and previous studies suggest that low temperatures can induce the transcriptional upregulation of nitrate reductase [58,59]. Thangaraj et al. [60] discovered that nitrate reductase displayed maximum activity at 37 °C for both cold-tolerant and warm-tolerant strains of the cyanobacterium *Nostoc*. Additionally, the cold-tolerant strain showed maximum glutamine synthetase activity at 4 °C, while the warm-tolerant strain showed maximum activity at 37 °C [60]. These results suggest that nitrogen fixation/assimilation enzymes collaborate through distinct mechanisms at varying temperatures for nitrogen exploitation. However, in our study, nitrate transporter gene (*Nrt*) and nitrate reductase gene (*Nr*) were downregulated under LT storage compared to the NT storage condition (Supplementary Figure S4a). At low temperatures, the expression level of the gene encoding formamidase decreased, resulting in a reduction in the conversion of ammonia and formic acid. In addition, glutamate dehydrogenase (*gdhA*), glutamine synthetase (*glnA*), and glutamate synthase (*gltS*) were also downregulated in the LT group, indicating that the formation of ammonia and its conversion to l-glutamate were suppressed under low temperature conditions.

On the other hand, in the HT group, nitrite reductase gene (*nirA*), encoding an iron oxidoreductase protein, was downregulated, which weakens the reduction in nitrate to ammonia. Conversely, there was an upregulation of the glutamate dehydrogenase (*gdhA*), glutamine synthetase (*glnA*), and glutamate synthase (*gltS*) genes were upregulated (Supplementary Figure S4b), which promotes the conversion of ammonia. However, the lack of ammonia in cells under high temperatures inhibits the synthesis of amino acids and other important compounds. In general, low expression of redox-related genes and high expression of oxidative genes under LT and HT storage may lead to the accumulation of ROS, which could contribute to cell death.

The fatty acid desaturase enzymes Δ9 and Δ12 are rate-limiting enzymes involved in the generation of oleic acid (18:1, Δ9) and palmitoleic acid (C16:1), respectively [61,62]. In this study, the genes encoding the desaturases ∆9, ∆12,3-Oxoacyl-[acyl-carrier-protein] reductase, 3-Hydroxyacyl-CoA dehydratase, and Peroxisomal-enoyl-CoA reductase in the unsaturated fatty acid pathway were all found to be downregulated in the LT group compared to the NT group (Supplementary Figure S5a). These results suggest that low temperature may inhibit the synthesis of unsaturated fatty acids by inhibiting the synthesis of oleic acid and linoleic acid, and also reduce the antioxidant capacity of *O. borgei* Chlococcus. Nevertheless, genes encoding the ∆9 and ∆12 desaturases 3-Hydroxyacyl-CoA dehydratase and Peroxisomal-enoyl-CoA reductase were all up-regulated in the HT group compared to the NT group (Supplementary Figure S5b), indicating that high-temperature stimulation promoted adaptation to high temperatures via alterations in polyunsaturated fatty acids in *O. borgei.*

Previous studies indicated that low temperature promotes the accumulation of polyunsaturated fatty acids in microalgae [63,64]. In this study, low temperature did not activate the synthesis of polyunsaturated fatty acids, while high temperature stimulation increased the activity of polyunsaturated fatty acid synthesis. This difference among studies suggests that the effect of temperature on the degree of unsaturation of fatty acids is highly species-specific. A temperature of 5 °C failed to induce excessive synthesis of polyunsaturated fatty acids as a defense mechanism against cold damage, owing to the broad temperature tolerance exhibited by *O. borgei.* Instead, low temperatures led to a decrease in the activity of some enzymes, inhibiting the polyunsaturated fatty acid synthesis pathway. However, under high-temperature conditions, *O. borgei* increases unsaturated fatty acid production through upregulation of oxidative genes involved in nitrogen metabolism. Further investigations are warranted to explore the specific types of unsaturated fatty acids and ROS levels during storage.

## 4. Materials and Methods

### 4.1. Cultivation, Concentration, and Preservation of O. borgei

*O. borgei*, supplied by our laboratory, was cultured for 14 days with f/2 medium (at a salinity of 30) under conditions outlined by Huang et al. [29]. Following a 24 h sedimentation period, the microalgae cells were concentrated and subsequently sealed in a normal sterile plastic bottle with a 100 mL volume, at a concentration of 1.19 × 10^7^–1.21 × 10^7^ cell/mL, with 80 mL per bottle. The concentrated microalgae solution was stored in light incubators (Ningbo Prandt Instrument Co., Ltd., Ningbo, China) at 5 ± 1 °C, 25 ± 1 °C, and 35 ± 1 °C for 50 days, respectively, with three parallel bottles for each temperature group. The light cycle was set for 12 light/12 dark, and the light intensity was kept at 30 μmol·m^−2^·s^−1^. During the 50-day storage, microalgae were shaken once every 5 days.

### 4.2. Determination of Polysaccharide and Lipid Contents

A 5 mL sample of microalgae solution was taken every 10 days to determine the contents of polysaccharides and lipids. The polysaccharide content was determined using the phenol-sulfuric acid method [65]. A glucose (analytical pure) standard curve was established at 20, 40, 60, 80, and 100 μg/mL concentrations, with the glucose concentration as the abscissa and optical density (OD) as the ordinate. After sample treatment, absorbance at 490 nm was measured using the EnSpire multilabel plate reader (Agilent Technologies, Inc., Santa Clara, CA, USA), and the polysaccharide content was calculated according to the regression equation for the standard curve. Lipid contents were determined based on previously described methods [66] with slight modifications. After treatment with a 20% dimethyl sulfoxide aqueous solution, staining was performed and the fluorescence intensity at a wavelength of 570 nm was measured using the EnSpire multilabel plate reader (PerkinElmer, Inc., Waltham, MA, USA), with an excitation wavelength of 480 nm.

### 4.3. Determination of the Cell Survival Rate and Recovery Efficacy

The cell survival rate was determined using the nucleic acid stain SYTOX Green (Thermo Fisher Scientific (Shanghai, China) Co., Ltd., Catalog number: S7020), with slight modifications to the method described by Zetsche and Meysman [67]. Briefly, 1 mL of microalgae solution was collected after storage for 50 days and centrifuged at 5000 rpm for 5 min. The culture medium was discarded, and the samples were washed twice with 1× PBS, followed by light-avoiding staining using 1 mL of 20 mM SYTOX Green for 20 min. Microalgae were observed using a fluorescence microscope (Olympus BX53, Tokyo, Japan). Argon ion was used to excite SYTOX Green, with an excitation wavelength of 488 nm and an emission wavelength greater than 523 nm. Dead cells exhibit green fluorescence (SYTOX Green positive), while live cells exhibit no fluorescence. The survival rate is expressed as the percentage of cells in the field of view that do not exhibit green fluorescence. After 50 days of storage, 5 mL of microalgae solution was taken and transferred to a 250 mL conical flask containing 95 mL of fresh f/2 medium. The cell density was measured at room temperature for 10 consecutive days and the specific growth rate was calculated using the following formula: K = (lnAx−lnA_X0_)/(d_x_−d_X0_), where A_x_ represents the cell density on day d_X_, A_X0_ represents the cell density at the initial time point, and d_X_−d_X0_ represents the time interval.

### 4.4. RNA Extraction, cDNA Library Construction, and Sequencing

On day 50, all remaining microalgae liquid from each bottle was placed in a 50 mL centrifuge tube, then the microalgae samples were centrifuged at 10,000× *g* for 10 min and the supernatants were discarded. The microalgae were washed twice with 1× PBS and then centrifuged to obtain microalgae pellets. Then, the microalgae pellets were ground into powder with liquid nitrogen and total RNA was extracted according to the manufacturer’s instructions of RNAprep Pure Plant Plus Kit (TIANGEN, Beijing, China; Product ID: DP441). After passing quality control tests for concentration, purity, and integrity, the total RNA was sent to a sequencing company (Guangzhou Kidio Biotechnology Co., Ltd. Guangzhou, China) for cDNA library construction, sequencing, and analysis. The sequencing platform was the Illumina HiSeq 4000. All raw data were submitted to the China National GeneBank Database (CNGBdb) under the accession number CNP0004218.

### 4.5. RNA-Seq Analysis

High-quality clean data for subsequent assembly and analysis were obtained from raw data using fastp [68]. Subsequently, the clean reads were assembled using Trinity [69], resulting in unigenes. The completeness of the assembly was evaluated using BUSCO [70], and unigenes were compared and functionally annotated by BLAST searches against databases, such as Non-Redundant Protein Sequence Database (Nr), Cluster of Orthologous Groups of proteins (COG), Swiss-Prot, and Kyoto Encyclopedia of Genes and Genomes (KEGG) [71]. Gene expression levels were calculated using the RPKM method, and a principal component analysis (PCA) was performed using R (v 3.6) based on these gene expression levels. An ellipse with a 95% confidence interval was plotted for visualization. DEGs were identified using DESeq2 [72], with FDR < 0.05 and |log_2_FC| > 1 as thresholds for significance. DEGs were further evaluated by gene ontology (GO) and KEGG enrichment analysis.

### 4.6. Real-Time Quantitative Polymerase Chain Reaction (RT-qPCR) Validation

Ten DEGs were selected for validation. Primers (Supplementary Table S11) were designed using Primer5 based on assembly results, and the accuracy of RNA-Seq results was verified using RT-qPCR. cDNA was synthesized using the PrimeScript RT Reagent Kit with gDNA Eraser (Takara, Beijing, China). The reaction system was configured according to FastStart Universal SYBR Green Master (ROX), and amplification and detection were performed using the LightCycler 96 real-time detection system (Roche, Forrentrasse, Basel, Switzerland). The PCR conditions consisted of preincubation at 95 °C for 30 s, followed by 40 cycles of denaturation at 95 °C for 10 s, annealing at 60 °C for 30 s, and extension at 72 °C for 30 s. The melting curve was plotted according to the instrument’s default parameters, and all samples were evaluated by three technical repeats. The 2^−ΔΔCT^ method [73] was used to calculate the relative expression of target genes, and log_2_FC values were obtained.

### 4.7. Statistic Analysis

Statistical analysis of data for polysaccharides, lipids, cell density, and specific growth rate of *O. borgei* were conducted using GraphPad Prism 9 (GraphPad Software, LLC, San Diego, CA, USA). Polysaccharide and lipid contents were tested by two-way ANOVA and Tukey’s multiple comparison tests, while cell density and specific growth rate were tested using one-way ANOVA. All analyses were performed at a confidence level of alpha = 0.05, and other parameters were set to default values. In addition, the microalgae cell survival rate was analyzed using *t*-tests implemented in GraphPad Prism 9 with a confidence level of alpha = 0.05 and all other parameters set to default values.

## 5. Conclusions

**Figure 6:**
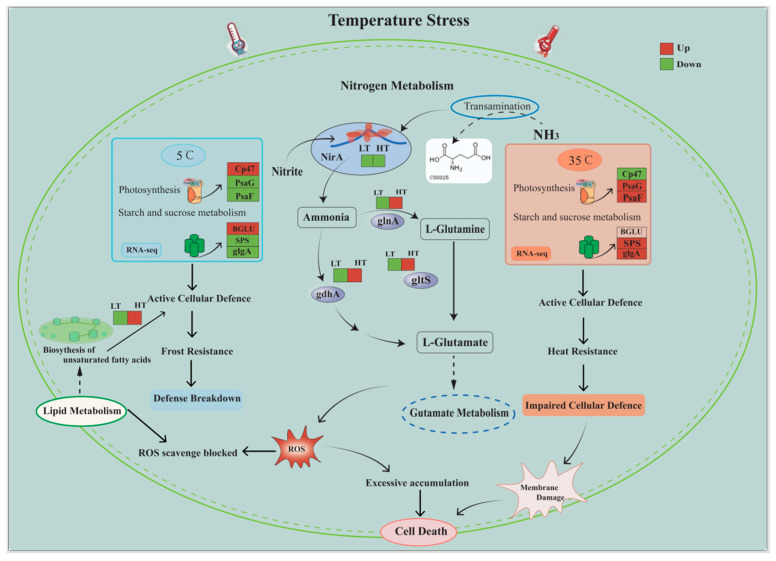
*O. borgei* transcription model under low or high temperature storage conditions.

This study demonstrates that the survival rate and resuscitation of concentrated *O. borgei* stored at room temperature surpassed those stored at both low and high temperatures. *O. borgei* adapts to temperature fluctuations by regulating photosynthesis, nitrogen metabolism, and the synthesis of polyunsaturated fatty acids. However, algal cells die due to excessive nutrient consumption during long-term storage. In the LT group, the downregulation of *PsaD*, *PsaE*, *gdhA*, *glnA*, and *gltS* in *O. borgei* (Figure 6) may lead to the inhibition of ATP synthesis and reduced efficiency in ammonia conversion. This could potentially account for the lower preservation efficiency observed compared to that in the room temperature group. In the HT group, *O. borgei* accumulates starch and trehalose by upregulating the expression of *UPG2* and *PGM*, enhancing thermotolerance. However, low expression of redox-related genes (*nirA*) and the overexpression of oxidative genes (*gdhA*, *glnA*, and *glts*) caused the accumulation of ROS (Figure 6), resulting in poor storage outcomes. In summary, the findings suggest that room temperature (25 °C) is the ideal storage condition for concentrated *O. borgei*, and the study provides theoretical support for the development of *O. borgei* as a live ecological agent for aquaculture.

## Data Availability

All data generated or analyzed during this study are included within the article and supplementary materials. The raw data are available from the corresponding author upon reasonable request.

## Supplementary Materials

The following supporting information can be downloaded at: https://www.mdpi.com/article/10.3390/ijms242216225/s1.
