# Supplementary material for: Transcriptome Analysis Revealed the Advantages of Room Temperature Preservation of Concentrated Oocystis borgei Cultures for Use in Aquaculture
Source: Int J Mol Sci. 2023 Nov 12;24(22):16225. doi: 10.3390/ijms242216225 (PMC10671278; doi:10.3390/ijms242216225)
Supplement: Supplementary file 1 [file ijms-24-16225-s001.zip › Supplementary figures.pdf]

Supplementary Figure S1. Nr species comparison chart of *Oocystis borgei* RNA-Seq

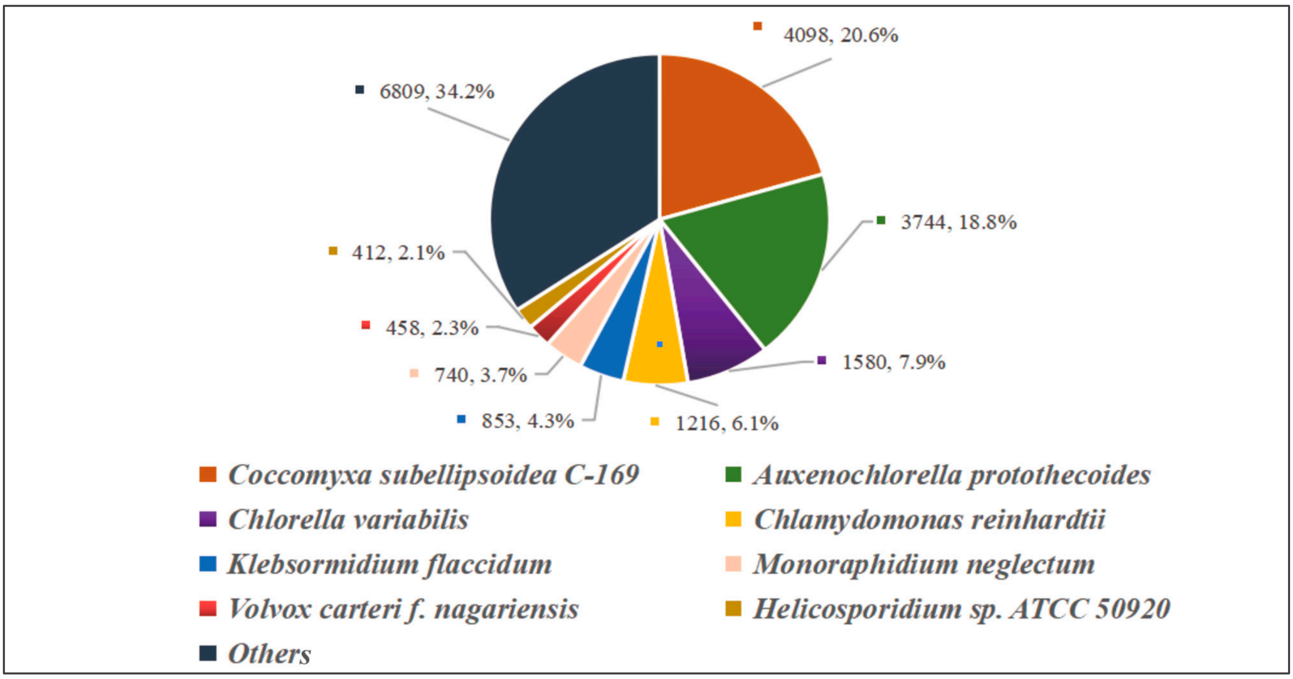

Supplementary Figure S2. Melting curve of RT-qPCR primer

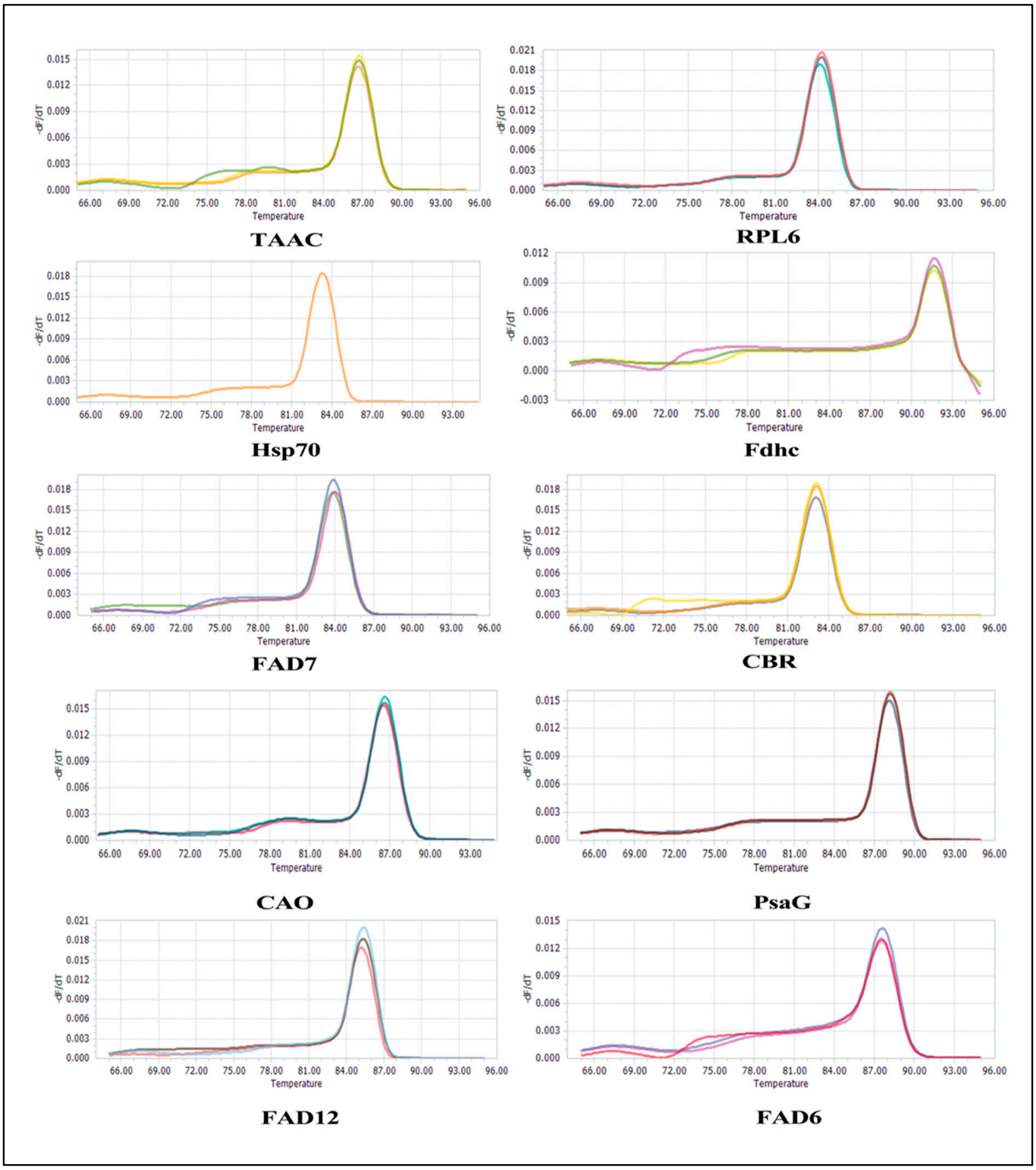

Note: 3 technical repetitions for each primer pair

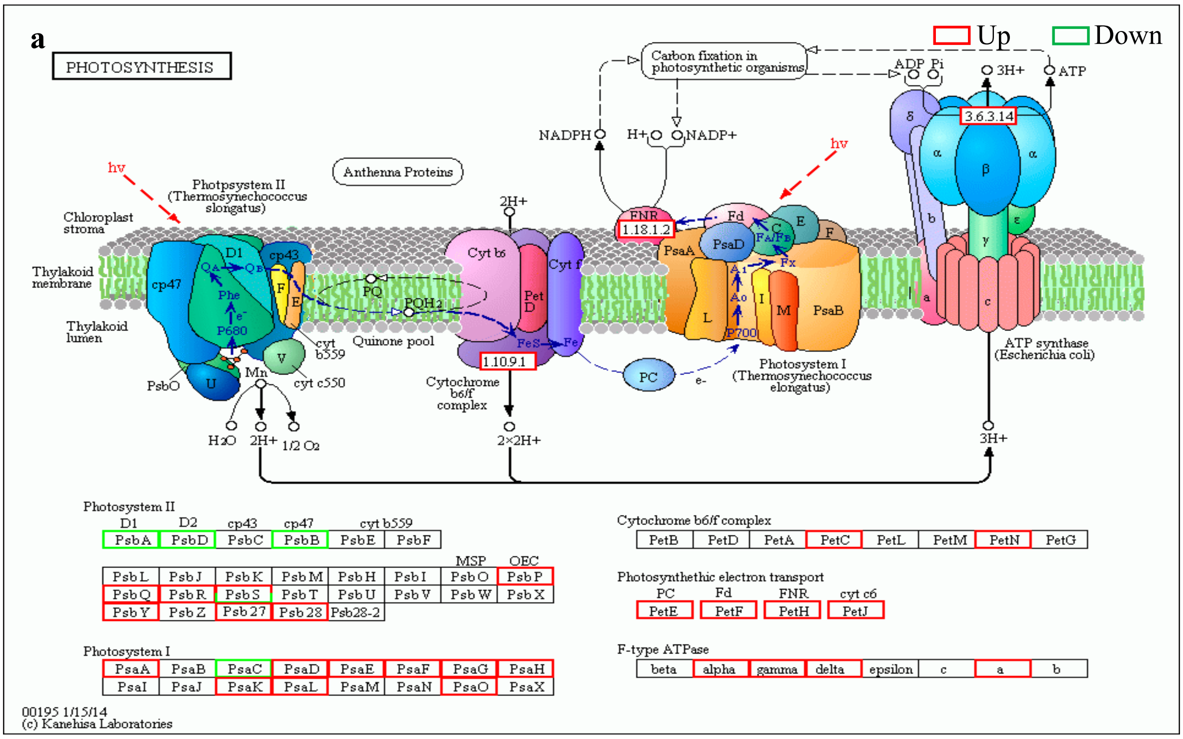

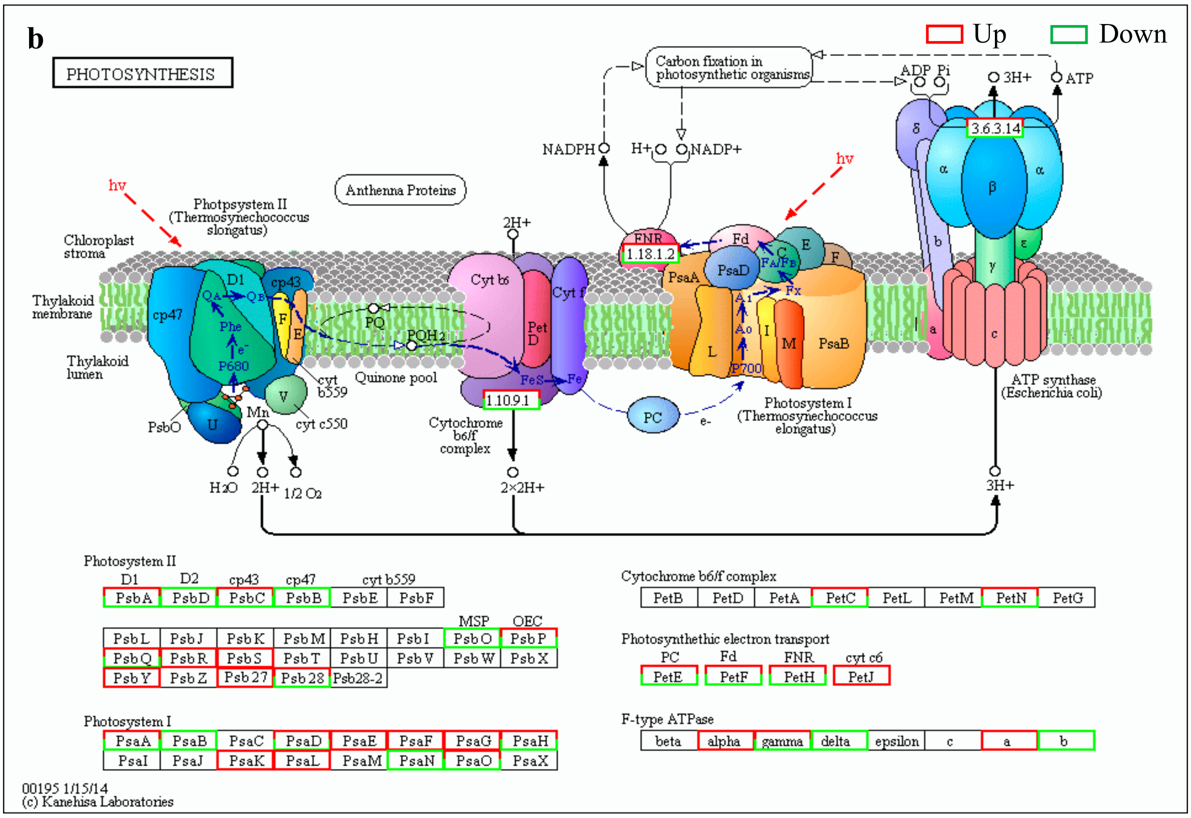

Supplementary Figure S3. Photosynthesis (a) LT vs NT; (b) HT vs NT

Note: (“A vs B” is B normalized to A)

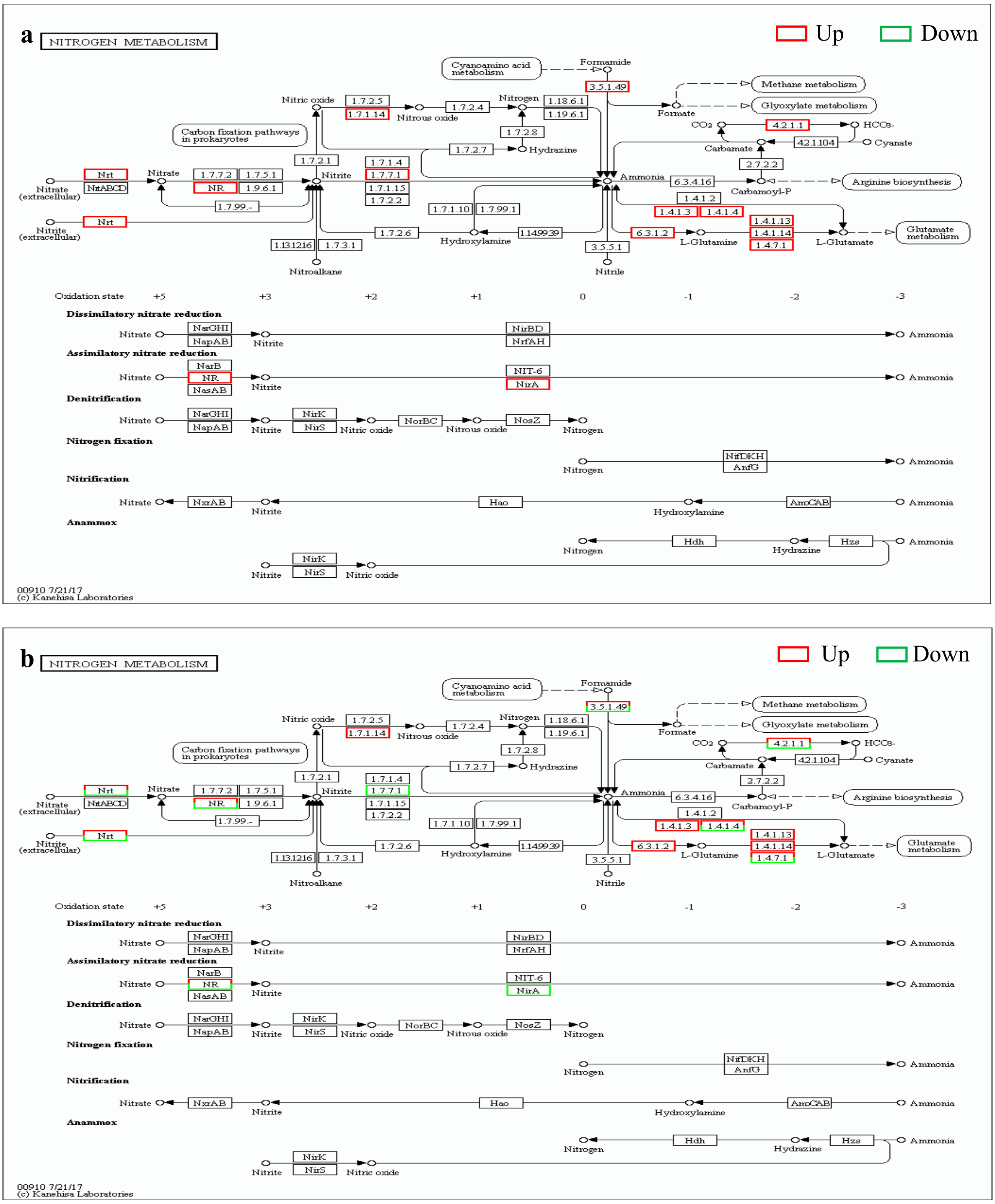

Supplementary Figure S4. Nitrogen metabolism pathway **(a) LT vs NT; (b) HT vs NT**

Note: (“A vs B” is B normalized to A)

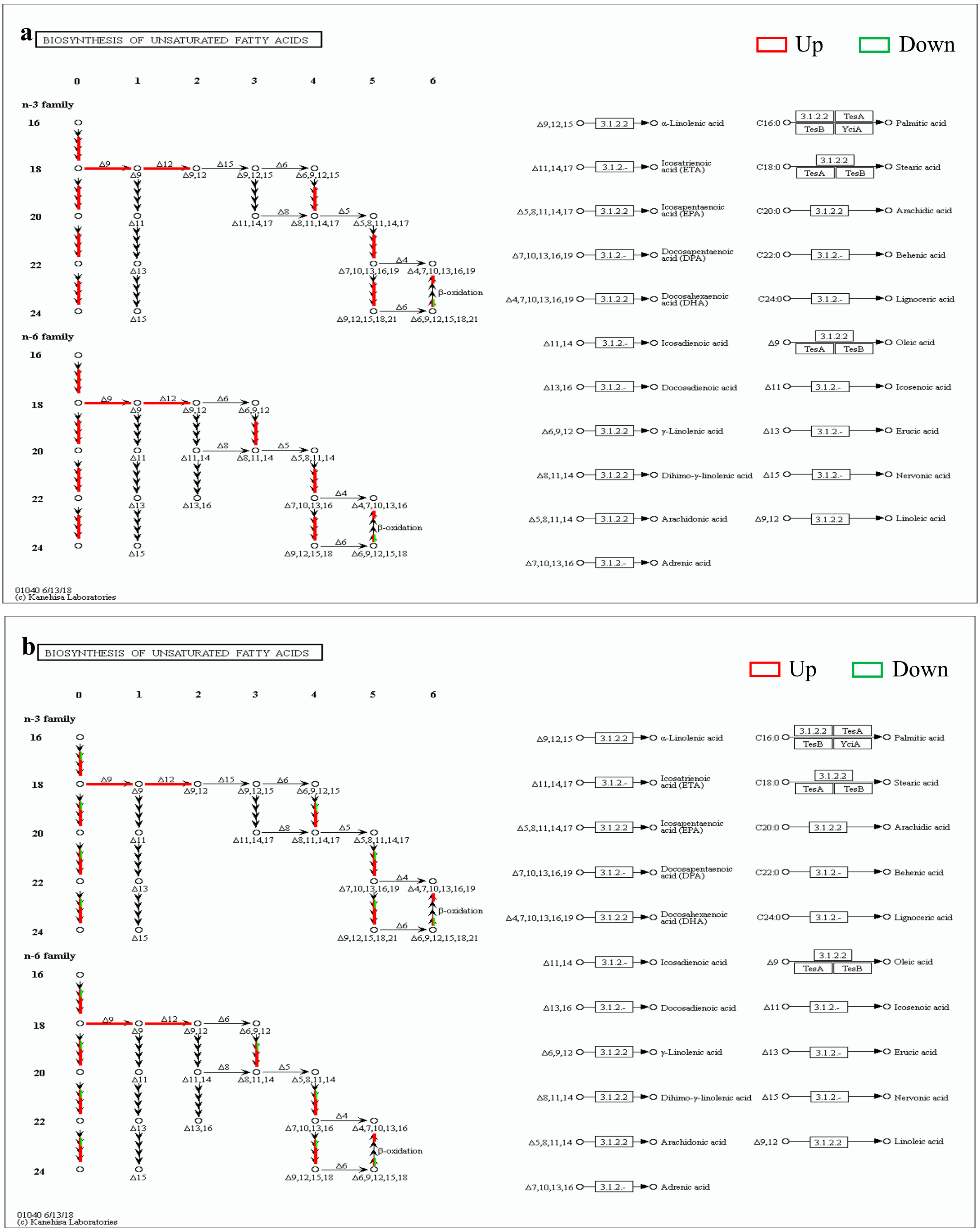

Supplementary Figure S5. Unsaturated fat acid synthesis pathway (a) LT vs NT; (b) HT vs NT

Note: ("A vs B" is B normalized to A)
